# Efficacy of levetiracetam, lamotrigine and sodium valproate on seizure attacks and EEG disorders in patients with juvenile myoclonic epilepsy: A double blind randomized clinical trial

**DOI:** 10.22088/cjim.13.3.617

**Published:** 2022

**Authors:** Sajjad Daneshyar, Masoud Ghiasian, Sahar Moradi, Elham Khanlarzadeh

**Affiliations:** 1 Department of Neurology, School of Medicine, Hamadan University of Medical Sciences, Hamadan, Iran.; 2 Department of Community Medicine, School of Medicine, Hamadan University of Medical Sciences, Hamadan, Iran

**Keywords:** Sodium valproate, Levetiracetam, Lamotrigine, Juvenile myoclonic epilepsy

## Abstract

**Background:**

Juvenile myoclonic epilepsy (JME) is one of the most important types of generalized idiopathic epilepsy. Patients generally respond quickly and perfectly to standard antiepileptic drugs but lifelong medication is necessary. Sodium valproate is the drug of choice in most references but it has some adverse reactions and some patients cannot tolerate the complications. Because of the need for life long treatment in this young aged group particularly child bearing women, we aimed to analyze the efficacy of these drugs to determine which has better efficacy with lower adverse effects.

**Methods:**

In this double-blind clinical trial 102 patients suffering from juvenile myoclonic epilepsy were randomly divided to three groups and treated with valproate, levetiracetam or lamotrigine and followed for 12 months at specified intervals.

**Results:**

Patients' mean age was 22.8 years and 28.4% of them were males and 71.6% were females. Effective terminal dose of sodium valproate, levetiracetam and lamotrigine were 1000, 1000 and 250mg, respectively. The rate of failure in controlling seizures and myoclonic jerks in lamotrigine group was meaningfully more than levetiracetam and sodium valproate (P=0.037).The general side effects of sodium valproate were much more; but there was not any significant difference between their effects on electroencephalogram (EEG) findings (P=0.81).

**Conclusion:**

Levetiracetam and sodium valproate have similar efficacy. But in the group of lamotrigine, rate of failure, myoclonus and drug adverse reactions were meaningfully more than sodium valproate and levetiracetam. According to our study, lamotrigine could not be a suitable treatment option for JME patients as a mono therapy. Levetiracetam can be a good alternative to sodium valproate, especially in women of childbearing age.

In 2016, there were 45.9 million patients with all-active epilepsy (both idiopathic and secondary epilepsy globally; age-standardized prevalence 621·5 per 100 000 population (1).The incidence of epilepsy in the US, is estimated 44 people per 100,000 persons each year .It has also been estimated that slightly less than 1percent of persons in the United States will have epilepsy by the age of 20 years(2). One of the most important types of idiopathic generalized seizures, is juvenile myoclonic epilepsy (JME). This kind of epilepsy is seen among child and young adults and consists of myoclonic jerks, generalized tonic clonic seizures and sometimes absence attacks (3). The mean age of patients is 15 years(8-34years).Characteristically these attacks specially myoclonic jerks worsen in the early morning (3, 4).

Imaging findings of the brain are normal. The electroencephalogram-gram (EEG) shows generalize spikes and irregular poly spikes of 4-6 Hz (5).The diagnosis is based on history of generalized tonic clonic seizures, myoclonic jerks and EEG findings (3, 4). The underlying cause of JME is not known and there are likely complex underlying genetic defects. Seizures will relapse by stopping the treatment in more than 80% of cases. Patients generally respond quickly and perfectly to standard antiepileptic drugs. But lifelong medication is essential and seizures will relapse in most of cases following stopping the drug (6).Sodium valproate which facilitates GABA activity, is the drug of choice in most references .In case of sodium valproate intolerance or not having proper response, lamotrigine, topiramate, zonisamide or levetiracetam can be used (7). Lamotrigine functions by selectively blocking the slow sodium channel. Levetiracetam may be the best new drug against seizures in treatment of juvenile myoclonic epilepsy, it affects the SV2A synaptic vesicle protein noticing the fact that it is well tolerated and has few complications, so it can be an alternative for sodium valproate in treatment of this disease(8) Because of the need for lifelong treatment in this young-aged group particularly child bearing women, we aimed to analyze the effectiveness of these drugs (valproate sodium, levetiracetam, lamotrigine) to determine which has better efficacy with lower side effects.

## Methods

This study was a double-blind control trial. The trial is registered at Iranian Registry of Clinical Trials, number IRCT201509079014N79 Registration date: 2015-09-18. We chose JME patients from referrals to Emam Neurology Clinic of Hamadan from 2013 to 2016. They were systemically examined by standards that were revealed by International League against Epilepsy. We chose newly diagnosed patients (no previous drug history) and excluded those with these criteria: having serious organic and metabolic disease (heart, hepatic, renal failure), age under 12 years, severe laboratory and electrolyte disturbances, MRI abnormalities, the presence of an underlying cause for the seizure, mental retardation, pregnancy and lactation period, alcohol or drug dependency, and BMI under 18 or over 40. Randomly patients were taken under treatment of levetiracetam, sodium valproate or lamotrigine. At the first step, biochemistry tests, EEG and MRI (based on exclusion criteria) were taken. Before starting the treatment, sequences of research were explained completely and patients were informed about the drug complications. All EEGs were taken by one standard EEG machine “Siemens Nihon –Kohden” in a standard room and were analyzed by one specialist and patients were advised to avoid sleeping prior time to each visit. Each EEG record was taken within 20-30 minutes and include at least 3 montages. We observed 102 patients on 1th, 2th, 6th, 12th, month of treatment and EEG, lab test were repeated in each visit. Numbers of seizures (GTC & myoclonic jerks) in the past month and drug adverse reactions were recorded in the adjusted questionnaire. The treatment started with the dosage of sodium valproate (Depakin brand) of 500 daily, levetiracetam (Cobel Darou Company) 500mg daily and lamotrigine (Sobhan Daru Company) 100mg daily. Then the dosage was gradually increased until seizures became under control or drug complications appeared or the drug reached its full dosage (sodium valproate 3000mg daily, levetiracetam 3000mg daily and lamotrigine 500 daily). 

The terminal effective dosage was recorded. Drug dosage for adults based on the text books are: 1000-3000 for valproic acid, 300-500 for lamotrigine and 500-3000 for levetiracetam. At the end of the study and after 12 months, by comparing the frequencies of seizures before and after treatment in the three groups, the results were analyzed with Kruskal Wallis test, ANOVA and independent t-test for each group and the effectiveness of drugs was compared. For better and more accurate evaluation, patients were analyzed for treatment outcome in three groups (defined by researcher);1.Drug failure: means uncontrollable seizures even with maximum dosage or serious or uncontrollable adverse reactions.2: patients with good response to the drug ; with completely controlled episodes of GTC reduction of myoclonic jerks to less than 50% comparing to pretest condition and having two normal EEG in row for last two visits.3.moderate responders means patients without any GTC episodes and myoclonic jerks more than 50% from the base line and/or still having abnormal EEG in two last appointments .

The study was conducted under the auspices of the ethical board of Hamadan University of Medical Sciences and registered with the number IR.UMSHA.REC.1394.71 in the Research Ethics Committee of the University of Medical Sciences.

## Results

From 108 participants in this study, 6 patients were out of the study because they did not use drugs carefully or they did not come for visits in determined times. Eventually, we had 41 persons in sodium valproate group and 31 persons in levetiracetam group and 30 patients in lamotrigine group. The elimination of mentioned patients did not have any influence on statistical result of the research. There was not any meaningful difference in the mean age, gender, body weight and the mean GTC and myoclonus frequency at the beginning of study between these three groups in independent t-test. Table 1 shows the demographic specifications of participants of this study. And table 2 shows the mean of myoclonic jerks and GTCs frequencies, monthly before and after treatment. (tables 1, 2). Table 3 shows treatment outcome in these three groups based on designed criteria that mentioned in methods section. After one month treatment ,the mean myoclonic jerks reduced from 14.61 to 1.66 in sodium valproate group(about 82% reduction), from 11.23 to 1.35(about 87% reduction) in levetiracetam group and from 16.37 to 4.63(about 71% reduction) in lamotrigine group, so the number of myoclonic jerks in lamotrigine group was significantly higher than sodium valproate and levetiracetam group. And the mean generalized tonic-clonic attacks were controlled in sodium valproate group from 0.93 to 0.03 in levetiracetam group from 1.33 to 0.19 and in lamotrigine group from 1.30 to 0.56 after 3 months of treatment. That means more than 96% of patients in sodium valproate group and more than 85% in levetiracetam and more than 56% in lamotrigine group were controlled. So, in our study about 96% of tonic clonic attacks and about 87% of myoclonic jerks were controlled.

**Table 1:** Demographic features of participants

**Gender**	**Male** **N=29 (28.4%)**	**Female** **N=73 (71.6%)**
Age	Range=14–41 year	Mean=22.8 year (SD=6.58)

**Table 2:** Mean of myoclonic jerks and GTCs frequencies monthly, before and after the treatment

**Drug group**	**Myoclonic jerks before treatment**	**Myoclonic jerks after treatment**	**GTC before treatment**	**GTC after treatment**
Sodium valproate	14.6115.52	1.663.78	0.930.43	0.030.16
Levetiracetam	11.2311.9	1.355.36	1.331.84	0.190.48
Lamotrigine	16.3725.6	4.639.36	1.310.65	0.561.04
P-value	0.95 ^*^	0.045 ^*^	0.05 ^**^	0.005 ^**^

Kruskal Wallis test٭ AVONA^ **^

**Table 3:** Treatment outcome in three groups

**Drug group**	**Prevalence**	**Drug** **failure**	**Moderate response**	**Good response**	**P-value**
Sodium valproate	Number	3	32	6	0.035^*^
	Percent	7.3	78	14.7	
Levetiracetam	Number	2	25	4	
	Percent	6.5	80.6	12.5	
Lamotrigine	Number	9	16	5	
	Percent	30	50.3	16.7	
Total	Number	14	73	15	102
	Percentage	13.7%	14.7%	71.6%	100%

*chi-square test

According to the table, we can say that 92.7% of patients in sodium valproate group responded to the drug and 7.3% of patients did not. In levetiracetam group, 93.5% of patients responded and 6.5% did not. In lamotrigine group, 70% of patients responded and 30% of them did not. So, drug failure was significantly higher in lamotrigine group. There were not any significant statistical differences between sodium valproate and levetiracetam. But between these two drugs and lamotrigine, the difference was meaningful, and lamotrigine was less effective with more serious side effects. But in the general side effects of sodium valproate were much more relatively. Lamotrigine although had some positive effects in reducing jerks and GTC but it was accompanied with few effective results on the mean incidence of GTC and we had more failure (n=30%) with this drug. Any significant difference was not observed about treatment effect on EEG findings. In three groups (P=0.81) and in each group, EEG became normal in more than 80% of patients as show in table 4.

Table 5 shows most common adverse reactions and LFT abnormalities. As it shows, patients in sodium valproate group had more drug reactions and more abnormal LFT and these differences were meaningful with P=0.037.

**Table 4:** EEG findings before and after treatment

**Drug group**	**Normal EEG before treatment**	**Normal EEG after treatment**
	**N(%)**	**N(%)**
Sodium valproate	1(2.4)	34(94.4)
Levetiracetam	5(16.7)	24(96)
Lamotrigine	4(13.8)	22(91.7)
P-value	0.1 ^*^	0.81^*^

*chi-square test

**Table 5:** Top reported drug adverse reactions

Drug group	Complication							
	Rash N (%)	Memory impairment N (%)	Nausea N (%)	Hair loss N (%)	Obesity N (%)	Tremor N (%)	Dizziness N (%)	Abnormal LFT N (%)
Sodium valproate	0	25(61)	8(19.5)	19(46.3)	13(31.7)	15(36.6)	7(17.11)	11(26.83)
Levetiracetam	0	1(3.2)	5(16.7)	1(3.2)	2(6.5)	2(6.5)	9(29)	1(3.23)
Lamotrigine	5(16.7)	5(16.7)	8(26)	4(13.3)	2(6.7)	2(6.7)	6(20)	3(10)
Total	5(5)	31(30.4)	21(20.6)	24(23.53)	17(16.7)	19(18.6)	22(21.5)	15(14.8)

Serious side effects like rash were more frequent in lamotrigine group and adverse reactions of sodium valproate are usually reversible. P=0.037

## Discussion

One of the most important types of idiopathic generalized seizures is the juvenile myoclonic epilepsy (JME) (3). This type of epilepsy is the most common type of generalized idiopathic epilepsy specially in women (9) Because of stopping the medical treatment can lead seizures relapse in more than 80% of cases .Therefore ,there is a necessity for lifelong treatment for this disease (10). In our study, the mean age of patients was 22.8 years, which was 7.8 years higher than the average in other studies, which could be due to the number of patient samples. In this research, after 12 months of follow-up, the frequencies of generalized tonic colonic seizures and myoclonic jerks significantly decreased in all three groups,(sodium valproate, levetiracetam and lamotrigine) . Most cases were completely controlled. The mean GTC attacks after treatment were 0.03 for the group taking sodium valproate, and 0.19 for the levetiracetam group, and 0.56 for lamotrigine group. Comparison of GTC attacks and myoclonic jerks in three groups with ANOVA test shows meaningful differences between lamotrigine and other two drugs. (sodium valproate and levetiracetam had same effects without meaningful differences). In three groups (P=0.81), EEG became normal in more than 80% of patients. Also, the patients were analyzed for treatment outcome in three groups (defined by researcher as mentioned in methods). 92.7% of patients in sodium valproate group responded to drug and 7.3% of patients failed. In levetiracetam group, 93.5% of patients responded and 6.5% did not. In lamotrigine group, 70% of patients responded and 30% of them did not. In Scharpe’s study, levetiracetam (monotherapy) was prescribed for 30 JME patients and seizures were controlled in 80% of patients after a 6 month-follow-up and in 96.6% after 12 months (11).In another research, levetiracetam controlled 53.8% of myoclonus and 80% of generalized tonic colonic seizures (12). It was observed in another research that in 80% of patients taking levetiracetam, seizures were completely controlled (4). In Kashipazha’s research ,effectiveness of sodium valproate and levetiracetam were the same and they concluded that levetiracetam is a proper alternative treatment instead of sodium valproate which has appropriate effectiveness along with lower complications (13). Also,according to the result gained from M alharmarni’s study in 2016,levetiracetam was an appropriate choice for monotherapy in JME patients (14).

Our research also shows that in levetiracetam group, 93.5% of patients responded and 6.5% failed. The mean myoclonic jerks reduced, likewise in about 87% in levetiracetam group, 82% in sodium valproate group and 71%-lamotrigine group. So, both sodium valproate and levetiracetam drugs had meaningful effects on reducing the GTC attacks and myoclonic jerks after 12 months (P = 0.005 and 0.045 respectively). Because sodium valproate is contraindicated in pregnancy and according to our study most patients are females and of reproductive age, levetiracetam can be a good alternative to sodium valproate, especially in women of childbearing age (15). 

Also, in our study,there was no difference in seizure rate between non-pregnant women and men treated with sodium valproate and it had the same effect in both groups. Lamotrigine, although had some positive effects in reducing jerks and GTC, it was accompanied with few effective results and higher numbers of myoclonic jerks after treatment and also with serious side effects like rash (16.7%). In this research, there was not significant statistical difference between sodium valproate and levetiracetam but between these two drug and lamotrigine, the difference was meaningful.

 Limitations of this trial were lack of patients ‘cooperation during treatment , and inadequate numbers of qualified cases for entering the study, so we clearly explained the condition for the patients to gain the trust , we also extended the duration of the study , anyway, 5% sample shedding had been estimated before the study began .

**In conclusions a**s a result, levetiracetam was as effective as sodium valproate even with less side effects so it can be a good choice for replacing sodium valproate. And it can be a proper alternative treatment as first line medication for JME patient but for more accurate results, trials with bigger sample sizes and longer duration of follow-up is recommended. 

According to our study, lamotrigine could not be a suitable treatment option for JME patients as a mono therapy. Levetiracetam can be a good alternative to sodium valproate, especially in women of childbearing age.
